# Factors that influence the quality of metabolomics data in in vitro cell toxicity studies: a systematic survey

**DOI:** 10.1038/s41598-021-01652-1

**Published:** 2021-11-11

**Authors:** Marta Moreno-Torres, Guillem García-Llorens, Erika Moro, Rebeca Méndez, Guillermo Quintás, José Vicente Castell

**Affiliations:** 1 Unidad de Hepatología Experimental y Trasplante Hepático, Health Research Institute Hospital La Fe, Valencia, Spain; 2 Health and Biomedicine, LEITAT Technological Center, Barcelona, Spain; 3 Unidad Analítica, Health Research Institute Hospital La Fe, Avda Fernando Abril Martorell 106, 46026 Valencia, Spain; 4 Centro de Investigación Biomédica en Red de Enfermedades Hepáticas y Digestivas (CIBERehd), Instituto de Salud Carlos III, Madrid, Spain; 5 Departamento de Bioquímica y Biología Molecular, Universidad de Valencia, Valencia, Spain

**Keywords:** Drug discovery, Biomarkers, Diseases, Medical research

## Abstract

REACH (Registration, Evaluation, Authorization and Restriction of Chemicals) is a global strategy and regulation policy of the EU that aims to improve the protection of human health and the environment through the better and earlier identification of the intrinsic properties of chemical substances. It entered into force on 1st June 2007 (EC 1907/2006). REACH and EU policies plead for the use of robust high-throughput "omic" techniques for the in vitro investigation of the toxicity of chemicals that can provide an estimation of their hazards as well as information regarding the underlying mechanisms of toxicity. In agreement with the 3R’s principles, cultured cells are nowadays widely used for this purpose, where metabolomics can provide a real-time picture of the metabolic effects caused by exposure of cells to xenobiotics, enabling the estimations about their toxicological hazards. High quality and robust metabolomics data sets are essential for precise and accurate hazard predictions. Currently, the acquisition of consistent and representative metabolomic data is hampered by experimental drawbacks that hinder reproducibility and difficult robust hazard interpretation. Using the differentiated human liver HepG2 cells as model system, and incubating with hepatotoxic (acetaminophen and valproic acid) and non-hepatotoxic compounds (citric acid), we evaluated in-depth the impact of several key experimental factors (namely, cell passage, processing day and storage time, and compound treatment) and instrumental factors (batch effect) on the outcome of an UPLC-MS metabolomic analysis data set. Results showed that processing day and storage time had a significant impact on the retrieved cell's metabolome, while the effect of cell passage was minor. Meta-analysis of results from pathway analysis showed that batch effect corrections and quality control (QC) measures are critical to enable consistent and meaningful estimations of the effects caused by compounds on cells. The quantitative analysis of the changes in metabolic pathways upon bioactive compound treatment remained consistent despite the concurrent causes of metabolomic data variation. Thus, upon appropriate data retrieval and correction and by an innovative metabolic pathway analysis, the metabolic alteration predictions remained conclusive despite the acknowledged sources of variability.

## Introduction

There is a marked interest among in vitro toxicity researchers in developing robust high-throughput *omic* techniques for a better understanding of the underlying mechanisms of toxicity, improving global biomarker discovery, and more accurate hazard predictions. Great advances have taken place in the different “omics”, noteworthy metabolomics, thanks to the relatively recent technical improvements in the hyphenation of high-performance liquid chromatographic and mass spectrometric detection that enables the generation of large datasets of high-quality metabolomics experimental data as a valuable source for extracting useful biological information. Metabolomics aims at the comprehensive analysis of the complete set of small molecular weight metabolites (i.e., the metabolome) contained in a biological sample and the multi-parametric metabolic response of living systems to stimuli^1^. The metabolome represents the downstream products and interactions of genes, proteins, xenobiotics and environmental factors providing a functional readout of the status and metabolic performance of a biological system under study^2–5^, and so, an accurate functional description of the phenotype associated with its response to biological injury, diseases, or treatments, as well as for uncovering drug toxicity mechanisms^6–9^.

Metabolomics is widely used in biomedical research for the study of different biological samples (e.g. serum, urine, cells and tissues) and for goals including biomarker detection, the identification of altered metabolic pathways, disease diagnosis and toxicity assessments. Toxicity screening in the course of early drug discovery is of great relevance to safely select best drug candidates to minimize dropout failures in the following clinical stages. Assessment of the potential toxicity of chemicals in humans is also of increasing concern and relevance. In vitro cell systems have always been at the forefront of the 3R’s (Replacement, Reduction and Refinement of animal's tests) principles in toxicological studies^10^. These concepts are also being fostered by the EU REACH program aimed to provide animal and human relevant toxicity data, of a vast number of chemicals handled in the European Union with limited toxicological information, but without the use of animal experimentation^11^. Assays that were traditionally performed only in animals are nowadays precluded by in vitro experiments using human-relevant cellular models, with the aim of identifying human potential hazards and relevant mechanisms of toxicity, as well as to anticipate interspecies differences and susceptibility in xenobiotic biodisposition and toxicity^11^.

Cellular metabolomics can provide valuable information on the mode of action of the chemicals tested, the altered outcome pathways (AOP’s), and toxicity mechanisms operating within cells exposed to a given compound^12–14^.

Metabolomic methods have long struggled to be a reproducible and generalizable analytical tool and its use in in vitro toxico-metabolomics is still hindered by several potential pitfalls. Furthermore, variation in metabolomic data resulting from pre-analytical and analytical sources (e.g., sample generation, collection, storage and preprocessing, batch effects, differences in chromatographic and MS performances) and biological sources (e.g., cell type and passage) need to be addressed and accounted for. In this context, diverse studies have already confronted some aspects as pre-centrifugation temperature, centrifugation forces, storage temperature, time and tubes, processing time, microclotting and hemolysis in blood and plasma samples^15–18^. Several initiatives, such as the ‘metabolomics standards initiative’ (Metabolomics Society), ‘COSMOS’, MetExplore or PhenoMenal have been carried out in the past recent years to establish minimum reporting guidelines, Quality Control (QC) and Quality Assessment (QA) procedures. However, these initiatives and recent guidelines^19^ do not cover a number of relevant experimental factors that concur in in vitro toxicology studies.

A number of cell culture features (e.g. cell-strain, cell passage number, cell sample collection, storage and processing), have a significant impact on the cell's metabolome and need further attention^19–23^. Although envisaged in the guidelines, when the analysis of a large number of samples is carried out, the so-called “within-batch effect” is always observed. In real life applications in in vitro toxicology research, studies are designed including large numbers of samples covering broad experimental conditions (e.g. different drugs, concentrations, incubation times) making unrealistic the use of a unique cell passage, the simultaneous processing of all samples, or even the sample analysis in a single analytical batch. All these factors represent sources of variability that unaccounted for, reduce the statistical power of the analysis.

The present study was designed to evaluate the impact of three selected experimental factors (cell passage, sample processing batch, and instrumental batch effects) and their interactions on the raw retrieved UPLC-MS metabolomic profiles, and on the results from functional metabolic pathway analysis in in vitro cell studies. For this purpose, differentiated human liver HepG2 cells at different passages were incubated with two known hepatotoxic compounds (acetaminophen and valproic acid) and a non-hepatotoxic compound (citric acid), and the cell extracts were processed in different batches and days. The analysis included uni- and multivariate analysis of the variations of intensity of annotated features, and the analysis of impact on the outcome from metabolic pathway analysis. Results showed that the experimental factor ‘cell passage’ had a lower impact on data than the sample processing batch and the storage time. Nonetheless, using metabolic pathway analysis of metabolomic data after within-batch effect correction, it was possible to properly and consistently identify meaningful metabolic pathway alterations and biological insights of the effects caused by xenobiotics despite the observed data variability.

## Material and methods

### Chemicals and reagents

Ultra-pure water was generated employing a Milli-Q Integral Water Purification System from Merck Millipore (Darmstadt, Germany). LC–MS grade solvents (CH_3_CN, isopropanol (IPA), and CH_3_OH) were acquired from Scharlau (Barcelona, Spain). Formic acid (≥ 95%), and ammonium acetate (≥ 98%) were purchased from Sigma-Aldrich Química SL (Madrid, Spain). Internal standards (ISs) phenylalanine-D_5_, tryptophan-D_3_ and caffeine-D_9_ were purchased from CDN Isotopes (Pointe-Claire, Canada). Valproic acid (VPA) (> 98%), acetaminophen (APAP) (> 99%) and citric acid (CA) (> 99%) were obtained from Sigma-Aldrich (San Luis, MI, USA).

### Cell culture and treatment of HepG2 cells

HepG2 cells (ECACC No.85011430) were routinely grown in culture grade flasks at 37 °C under a humidified atmosphere 5% CO_2_/95% air in Ham’s F-12/Leibovitz L-15 (1:1, v/v) supplemented with 7% fetal bovine serum (Capricorn Scientific GmbH, Ebsdorfergrund, Germany), 50 U/mL penicillin (Gibco, Waltham, MA, USA) and 50 μg/mL streptomycin (Gibco) as previously described^24–27^. Cells were ready to be used or passaged at 70–80% confluence. For subculturing purposes, cells were washed with Phosphate-Buffered Saline (PBS) and detached by treatment with 0.25% trypsin/EDTA (Thermofisher) at 37 °C.

This study was designed to assess the impact of the cellular passage and processing batch, and their interactions on the retrieved metabolomic profiles in toxico-metabolomic in vitro studies. Accordingly, a sufficient number of culture plates were foreseen in order to carry out the planned incubations in several batches but with the same cell passages in each batch. A vial of cells was thawed and for complete cellular recovery, cells were cultured and passaged for a week. After that, cells were seeded on testing plates at a density of 7.5 10^4^ cells/cm^2^ on 6-well culture dishes. Then, after 24 h, CA, APAP and VPA were added to culture plates at 1 mM and HepG2 cells were incubated for 24 h with the tested compounds. A meaningful toxicometabolomics assessment of tested drugs requires a concentration and incubation time high enough to cause alteration and harm to cells, but not excessive to induce extensive cell damage and lethality. According to previous work^27^, for the particular drugs used in our research 1 mM and 24 h incubation met the above-mentioned requirements that evidenced critical cellular events as oxidative stress without causing extensive cell death (ca. 10%).

Four biological replicates were generated for each assay condition. An additional plate with medium but without cells was included as blank.

**Figure 1:**
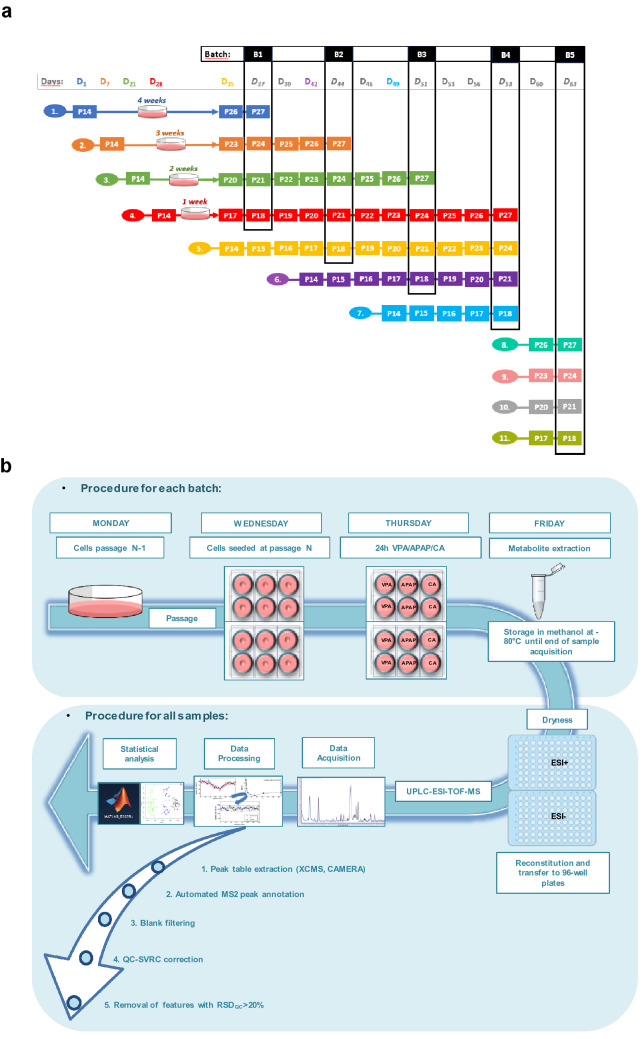
Experimental design. (**a**). Timing of the experiment, where D means day and the subindex indicates number of days elapsed since the beginning of the experiment. Processing batches (B1, B2, B3, B4 and B5) are shown in vertical black squares. Each batch was processed within 7 days of difference between each other, except for Batch 5 that was obtained 5 days after Batch 4. Each processing batch includes samples from four different passages (P18, P21, P24 and P27). After cell metabolite extraction, all cell extracts were stored at − 80 °C until final analysis in the UPLC-QqTOFMS. (**b**) Workflow of the experimental setup. Cells were thawed on day 1, 7, 21, 28, 35, 42 and 49. After subsequent passages, cells were seeded at the desired passage. 24 h later different treatments (CA, VPA and APAP) were independently added to the cells, and after 24 h cell treatment, samples were processed for metabolite extraction. Quadruplicate samples were analyzed for each experimental condition. Cell extracts were stored in methanol at − 80 °C until the end of sample collection. Afterwards, all samples were evaporated, reconstituted, and transferred to the 96-well plates and measured by UPLC-ESI-QqTOFMS on the same day. Data acquired was processed for peak table generation using XCMS and CAMERA software, metabolites were annotated by automated MS2 peak annotation, uninformative features were discarded by blank filtering and QC-SVRC correction and removal of features with RSD_QC_ > 20% was performed. Finally, statistical analysis was carried out for data analysis and interpretation.

The experimental setup is shown in Fig. 1a,b. It involved the incubation of HepG2 cells with two hepatotoxic compounds (APAP and VPA) and a non-toxic control compound (CA), using four different passage numbers (P18, P21, P24, and P27) under the same experimental conditions. Besides, HepG2 cells were processed in five batches (B1–B5), to evaluate the potential impact of the storage time of the cellular extracts in CH_3_OH at − 80 °C, prior analysis. An additional batch 5 (B5) was included as reference, in which cells were thawed at the desired passage and directly incubated with the xenobiotic, without being prior expanded as described above.

### Cell processing and metabolomic analysis

Cell processing was performed always in the same order to be consistent among batches. The culture medium was removed by aspiration after 24 h incubation with the xenobiotics. Cells were then washed with PBS at 37 °C, and then with PBS at 4 °C. 600 μL of ice-cold lysis solution (CH_3_OH:H_2_O (3:1), 0.125 μM ISs), were added to the 6-well plates for cell detachment and metabolite extraction. Plates were placed on dry ice to keep lysis solution cold. Then, cells were gently scraped from the plate, and the obtained cell extracts were transferred to an Eppendorf tube and placed on ice. The remaining cells in the plates were recovered by further addition of 200 μL of lysis solution (2×) washing and pooling all these volumes with previous cell extract volume (total volume 1 mL). Wells containing only media but no cells, as well empty wells, were extracted using the same procedure.

Three freeze–thaw cycles in liquid N_2_ were used to promote cell disruption and facilitate the metabolite extraction in the 1 mL cellular suspensions. Then, samples were centrifuged at 15,700×*g* (10 min, 4 °C) and supernatants were transferred to ice-cold Eppendorf tubes. Immediately after, samples were frozen in liquid nitrogen and stored at − 80 °C until all cell extracts from the whole experiment were collected.

Then, samples were evaporated to dryness under vacuum (SpeedVac). The remaining dry residues were re-dissolved in 70 μL of H_2_O:CH_3_CN (85:15, v/v), vortexed for few seconds, and centrifuged at 15,700×*g* (5 min, 4 °C) 30 μL of the supernatant were transferred twice to two 96-well plates for UPLC-QqTOFMS analysis in positive and negative ionization mode. A QC sample was prepared by pooling 10 μL of the supernatant from each processed sample.

Sample analysis was performed on an Agilent 1290 Infinity UPLC system from Agilent Technologies (Santa Clara, CA, USA) equipped with a Synergi™ Hydro-RP 80 Å LC (150 × 2 mm, 4 μm) column (Phenomenex, Torrance, USA). Further details are provided in Supplemental Material Procedures.

### Data pre-processing

Peak table generation was carried out for ESI^+^ and ESI^−^ independently using XCMS^26^. The *centWave* method was used for peak detection with the following parameters: mass accuracy = 25 ppm; peak width = (4,40) in ESI+ and (10,40) in ESI−; snthresh = 10; prefilter, (5,3000). A minimum difference in *m/z* of 10 mDa was selected for overlapping peaks. Intensity weighted *m/z* values of each feature were calculated using the *wMean* function. Peak limits used for integration were found through descent on the Mexican hat filtered data. Grouping before and after RT correction was carried out using the *nearest* method with 100 s as *rtCheck* argument, mzVsRT balance = 1 and kNN = 50 in ESI + and 25 in ESI-. Finally, missing peaks were filled by reintegrating the raw data files using the *fillPeaks* method and standard arguments. The XCMS CAMERA package^26^ was used for the identification of pseudospectra across samples using *xsAnnotate*, *groupFWHM*, *findIsotopes*, *groupCorr* and *findAdducts* using standard parameters. The accuracy of the peak table generation was assessed by comparing automated and manual integration results for ISs and a subset of endogenous metabolites, obtaining linear correlation coefficients higher than 0.98 (Fig. S1b). Metabolite annotation was carried out as described elsewhere^28^ by matching experimentally acquired MS/MS spectra with the predicted and experimental HMDB (www.hmdb.ca), METLIN^29^ and in silico LipidBlast^30^ databases. Further details about the annotation algorithm are provided in Supplemental Material Procedures.

Control blanks were employed to identify background features arising from the extraction solvents and mobile phase components and carry-over, and media blank samples were used to identify features arising from media contamination. Initial identification and elimination of these uninformative features was carried for ESI+ and ESI− data sets independently. LC–MS features where the ratio between the median peak area value in QCs and the 80% percentile of the distribution of peak areas observed in blanks was lower than 3, were considered as uninformative.

Within batch effect correction was carried out using the QC-SVRC approach employing a Radial Basis Function (RBF) kernel, as described elsewhere^25,27^. QC-SVRC requires the selection of three structural hyperparameters: the tolerance threshold (ε), the penalty term applied to margin slack values (C) and the kernel width ($\gamma$). The selection was carried out using a pre-selection of C and optimization of $\varepsilon$ and $\gamma$ using a grid search, leave-one-out cross validation and the RMSECV as target function. C was selected for each LC–MS feature as the median value of the peak areas observed in QCs. The $\varepsilon$ search range was selected based on the expected instrumental precision (2.5–8% of the median value of the distribution of peak area values in QCs). The $\gamma$ search interval selected was [1, 10^4^]. LC–MS features with D-ratio* > 20% were removed from further analysis, as described elsewhere^19^.

$$D - ratio_{i}^{*} = \frac{{MAD_{i,qc} }}{{MAD_{i,sample} }} \times 100 \%$$

Five samples were excluded from the analysis as they were considered as outliers given the low intensities in the total signal levels.

A total of 166 features were annotated in ESI+/− (89 and 77 measured in ESI+ and ESI−, respectively). Figure S1a shows the distribution of annotated features along the m/z-RT space, and summarizes the relative ratios of the main classes of annotated metabolites. The classes with the largest numbers of annotated metabolites were carboxylic acids and derivatives, purine nucleotides, glycerophospholipids and organooxygen compounds, accounting all together for 74% of the 166 annotated LC–MS features.

Schematic overview of the metabolomics analysis pipeline is shown in Fig. 1b.

### Data analysis and software

The data station operating software for data acquisition and manual integration was MassHunter Workstation (version B.07.00, Agilent). Raw data (.d) was converted into mzXML format using ProteoWizard (http://proteowizard.sourceforge.net/). Peak detection, integration, deconvolution, alignment and pseudospectra identification were carried out using XCMS (https://bioconductor.org/packages/release/bioc/html/xcms.html)^31^ and CAMERA (htpps://bioconductor.org/packages/release/bioc/html/CAMERA.html)^32^ in R 3.6.1 (https://www.r-project.org/^33^. Data analysis was carried out in MATLAB R2019a (Mathworks Inc., Natick, MA, USA) using in-house written scripts and the PLS Toolbox 8.7 (Eigenvector Research Inc., Wenatchee, USA). LipiDex open-source software^34^ was used to identify lipid species by matching the measured MS/MS spectra to an *in-silico* generated library (LipidBlast)^30^ using MS1 and MS2 searching tolerances set to 0.01 Da. Box and Whiskers were made with GraphPad Prism 6.0 (GraphPad software, San Diego,California USA, www.graphpad.com).

Principal Component Analysis (PCA) was carried out using autoscaled data. ANOVA Simultaneous Component Analysis (ASCA)^35^ was used to quantify the contribution of the distinct factors (cellular passage, processing batch and treatment) and their interactions to the overall variation. ASCA provides a multivariate ANOVA by applying a Simultaneous Component Analysis to each of the effects modeled by an ANOVA. Student’s *t* tests were used to test the null hypothesis that the continuous data in two groups (e.g. batch 1 vs. batch 2) comes from independent random samples with equal means and unknown variances, and *p* values < 0.05 were considered statistically significant.

The Pathway Analysis module on Metaboanalyst 5.0 website (http://www.metaboanalyst.ca/)^36^ was used using the metabolite peak intensities as input data. Data was log transformed and autoscaled, and it was matched against the human KEGG database^37–39^.

Raw data (.mzXML, .ms2) and peak tables used in this work are accessible via the Zenodo repository under https://doi.org/10.5281/zenodo.4971881.

## Results

### Within-batch effect analysis and correction

**Figure 2:**
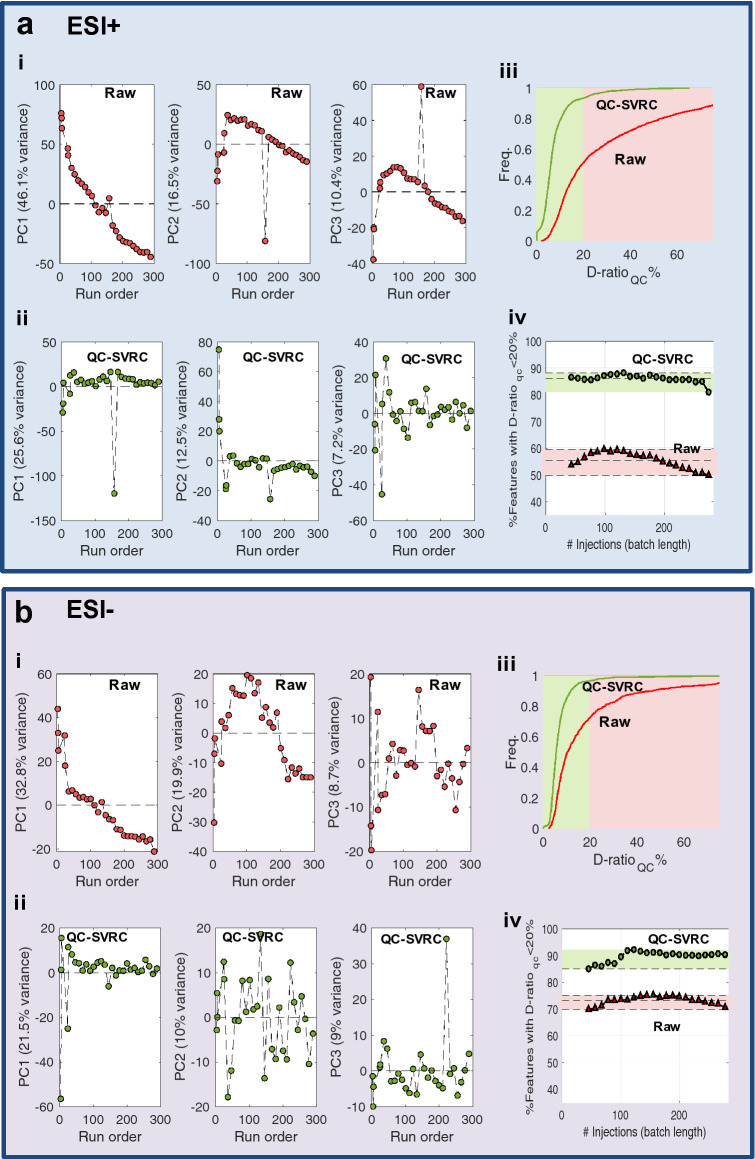
QC-SVRC correction eliminates within batch effect and improves data quality increasing the percentage of metabolites retained after data pre-processing. Results from ESI+ (**a**) and ESI− (**b**). PCA of the QC samples before (i) and after (ii) QC-SVR correction. Plots show the run order versus PC1, PC2 or PC3 and each dot represents a QC sample. The percentage of variance in each component is shown (% variance) (i, ii). Cumulative distribution functions of the D-ratio_QC_ % in the original dataset and after QC-SVRC (iii). Percentage of variables showing a D-ratio_QC_ < 20% in batches with different number of injections (iv).

Cell extracts of hepatocytes exposed to the three reference compounds were analyzed by reverse phase UPLC-QqTOFMS using ESI^+^ and ESI^-^ in independent analytical runs. In vitro toxico-metabolomic studies are designed to identify, frequently subtle, biological variations arising from the effect on an external intervention (e.g. xenobiotic), but variation in metabolomic data may result from both technical and biological sources as well, masking the effects of the compounds on cells. Changes in the instrumental response during the analysis samples should be detected and corrected in advance, to improve the data quality and facilitate obtaining reproducible outcomes. In our study, QCs were used for the identification and elimination of the within-batch effects^40^. Figure 2 shows the PCA scores plots calculated from the analysis of the QCs included in the ESI^+^ (Fig. 2a) and ESI^−^ (Fig. 2b) batches which were used to evaluate how the QC samples were clustered before and after batch correction. The decreasing trends in the scores plots as a function of the injection order evidenced that the within-batch effect was a relevant source of systematic error. PCA also identified QC#17 (injection order: 157) in the ESI^+^ batch as a potential outlier (Fig. 2a,b(i)). The use of QC-SVRC algorithm^41^ enabled the elimination of within-batch effect correction as shown in Fig. 2a,b(ii). The dispersion ratio (D-ratio*)^19^ was used to identify features with low precision, and to exclude them from further analysis. Figure 2a,b(iii) show the cumulative distribution functions of the D-ratio* values in QCs before and after QC-SVR correction. Before and after QC-SVRC, the proportion of features with a D-ratio < 20% were 49.9% and 70.7% (ESI+/−) versus 93.5% and 96.9% (ESI+/−) respectively, demonstrating that the QC-normalization increases considerably the number of features with a D-ratio < 20%. In addition, Fig. 2a,b(iv) displays the percentage of retained features using an acceptance criterion for the D-ratio set to < 20%. Results show that the improvement in the data quality and the number of retained features does not depend on the size of the analytical batch, providing a consistent increase in the relevant information which could be retrieved from the analysis, even for small batches (< 100 injections).

Besides batch effect elimination, data analysis workflow included the elimination of uninformative features to improve the precision and accuracy of the metabolic models and reduce the likelihood of serendipity correlations.

### Impact of the cell passage number, processing batch and bioactive compound treatment on cell’s metabolomic data

**Figure 3:**
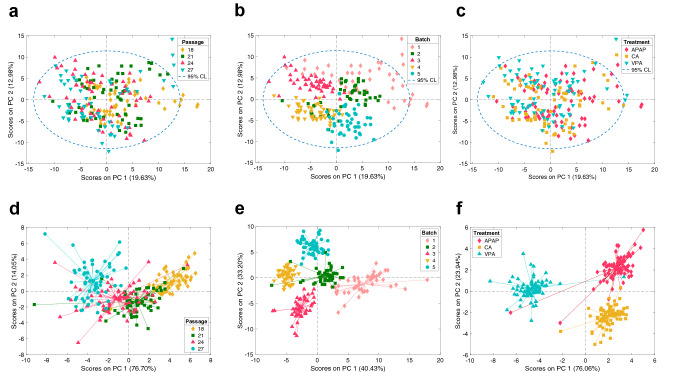
Multivariate data analysis of the influence of the cellular passage, processing batch and compound treatment by PCA and ASCA. Score plots from the analysis of 166 annotated metabolites based on cell passage (**a**), processing batch (**b**) and treatment (**c**). ASCA PC1 and PC2 scores plots for the factors ‘Passage’ (i.e. X_Passage_) (**d**), ‘Batch' (i.e. X_Batch_)’ (**e**), and ‘Treatment’ (i.e. X_Treatment_) (**f**).

PCA was used for an initial evaluation of the impact of the cell passage and the processing batch on the retrieved cell's metabolome data. Figure 3 depicts the PC1 *vs* PC2 scores of a PCA model accounting for 32.6% of the initial data variation. Results showed a high overlap among samples from different passage groups, indicating that cellular passage was not one of the main sources of variation in the retrieved data (Fig. 3a). On the other hand, the clustering of samples according to their processing batch indicated that differences associated to the processing day and the total time of storage until analysis were among the main sources of data variation (Fig. 3b). The random distribution of the processing batch clusters in the PCA scores space (Fig. 3a) and in the hierarchical clustering (see Fig. S2a) and the lack of linearity of the feature intensities with time (see Fig. S2b), suggested that this source of variability was mainly associated with the manual sample processing. ASCA was performed with the ANOVA model including 2-way interactions of three factors: X = Mean + X_Treatment_ + X_Batch_ + X_Passage_ + X_Treament-Batch_ + X_Treatment-Passage_ + X_Batch-Passage_ + E. Results presented in Table 1 showed a minor contribution to the variance caused by the passage number (6%), while the strongest effect was due to the processing batch, and was responsible for 27.6% of the total variation.

**Table 1:** Relative contributions of the effect of processing batch, cellular passage and drug treatment and their interaction to the total variation estimated by ASCA.

Term	PC	Effect (%)	*p* value
Passage	3	6.0	0.002
Batch	4	27.6	0.002
Treatment	2	8.7	0.002
(Treatment) × (Batch)	12	2.8	0.002
(Treatment) × (Passage)	10	1.1	0.598
(Batch) × (Passage)	18	14.3	0.002
Residuals		39.6	

*p values were estimated using 500 permutations. Effects were considered significant when the *p* value < 0.05.

Score plots of the two first PCs of the ASCA factor ‘passage’ and ‘processing batch’, are depicted in Fig. 3d,e showing the calculated effect of cell passage and the processing batch. Score plots of PC1 showed that differences between P18-21 with P24-27 were responsible for most of the cell passage associated variance (76.7%) (see Fig. 3d). Results depicted in Fig. 3e indicate that the distribution of sample batches was largely dominated by differences between Batches 1–2 and 3–4–5 along the first PC (40.4%). PCA also showed that the effects of the hepatotoxic compounds APAP and VPA were masked by the processing batch, and not included among the main sources of variance in the data set (Fig. 3c). However, results from ASCA confirmed the statistical significance of the factor ‘treatment’ on the data variation (Table 1). Scores of the two first PCs of the ASCA factor ‘treatment’ depicted in Fig. 3f, showed that samples incubated with any of the 3 compounds were separated among them revealing a different impact of these bioactive compounds on the cell's metabolome, independently of the cell passage or the processing batch. This highlights that an appropriate data analysis is capable of identifying subtle, but consistent metabolic changes induced by different compounds despite the above-mentioned sources of variability.

### Integrative evaluation of the effects of bioactive compounds on hepatocytes’s metabolome

**Figure 4:**
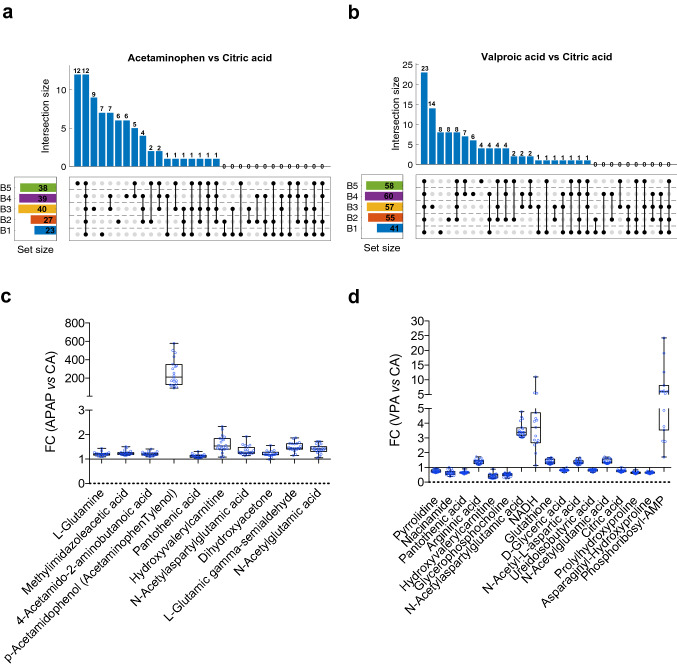
Analysis of the reproducibility of the metabolic alterations induced by xenobiotics. Upset plots to visualize the intersecting sets of metabolites that appeared statistically significant (*p* value < 0.05) in the *t* test analysis between acetaminophen and citric acid (**a**) or valproate and citric acid (**b**) from one up to five batches. Numbers on the set size squares indicate the metabolites that are statistically significant between compound comparisons in each batch. (**c**-**d**) Box plots of the fold change (FC) in the levels of the metabolites that were statistically significant in the *t* test between valproate or acetaminophen treatment versus citric acid in all five batches. FC > 1 means metabolite increase and FC < 1 decrease. Each dot represents the FC in a specific batch and passage.

We then examined the changes in the metabolome induced by two reference hepatotoxic substances (APAP and VPA) and compared with a non-hepatotoxic compound (CA) and verified the consistency and reproducibility of the biological outcome independently of the cellular passage and processing batch. For that purpose, we performed the identification of discriminant features by a *t* test analysis between APAP or VPA versus CA treated cells in each specific batch and cellular passage possible (20 different possible combinations batch/cellular passage, i.e., B1P18, B5P27). No significant metabolites on APAP and only Glycerophosphocholine in VPA treated cells appeared significantly altered in all conditions. Since our previous results demonstrated that passage number had only a minor influence on HepG2 cells retrieved metabolomic profiles, we selected the metabolites significantly altered (*t* test *p* value < 0.05) between APAP or VPA *vs* CA treated samples in each batch disregarding the cellular passage information. Figure 4a,b shows the number of metabolites that appeared altered as a function of the number of batches. The effect of the processing batch on the retrieved metabolic profiles reduced the repeatability of the analysis, as shown by the lower number of metabolites identified as altered in the 5 batches (12 (10 unique) and 23 (17 unique), in the APAP and VPA treated cells, respectively). To address the differences in the reproducibility of the changes associated with the experimental factors we calculated the fold change in the 10 and 17 metabolite levels induced by the hepatotoxic drug at each specific batch-passage combination (Fig. 4c,d). The direction of the fold changes was highly reproducible among all possible conditions tested, metabolite levels were either increased or decreased after the hepatotoxic drug treatment, and the intensity of the alteration was also greatly comparable. These results demonstrate that, given the unavoidable experimental factor influence that affects overall metabolite intensities, reproducible results can be obtained upon appropriate data analysis but caution is required regarding statistical repeatability since same effects are generally observed in different experiments but their magnitude or statistical significance can differ.

### Functional pathway analysis

We examined the perturbations observed in the metabolic pathways of hepatocytes incubated with the bioactive compounds. Thus, the analysis involved the comparison between two phenotypes. Then, metabolomic pathway analysis (MetPA) was then used to identify significantly altered metabolic pathways^42^ using, as enrichment method, the global test algorithm^43^ and as topology analysis the relative-betweenness centrality algorithm. The annotated features and their peak intensities were evaluated for each individual experiment, comparing APAP or VPA *vs* CA treatment. Pathway analysis was performed in Metaboanalyst for APAP *vs* CA or VPA *vs* CA comparisons for each batch. Six and nine metabolic pathways appeared significantly altered by APAP and VPA respectively in all batches (see Table S1). The significance of the correlation between results from functional metabolic pathway analysis obtained under different experimental conditions (e.g., the effect of APAP in two batches), was estimated using the Mantel’s test, the − log_10_(*p* value) and the impact factor as coordinates, the Euclidean distances as dissimilarity measure, and the Pearson correlation coefficient^44^. Table 2 summarizes the Mantel’s test^45^ coefficients and estimated significances (*p* values).

**Table 2:** Pearson linear correlation coefficient and *p* value from Mantel’s test obtained from paired comparisons of pathway analysis results. Pathway analyses were obtained comparing APAP or VPA versus CA in each batch. Correlation was performed comparing pathway analysis results from the same (APAP or VPA; left side) or different (APAP *vs* VPA; right side) treatments. One asterisk indicates *p* value < 0.05, two asterisks indicates *p* value < 0.01.

Comparison	Corr. coef	*p* val	Sig	Comparison	Corr. coef	*p* val	Sig
B1vsB2_APAP	0.12	0.109		B1_APAPvsB1_VPA	0.04	0.769	
B1vsB3_APAP	0.20	0.035	*	B2_APAPvsB2_VPA	0.24	0.023	*
B1vsB4_APAP	− 0.06	1.001		B3_APAPvsB3_VPA	− 0.05	1.001	
B1vsB5_APAP	0.16	0.035	*	B4_APAPvsB4_VPA	− 0.09	1.001	
B2vsB3_APAP	0.22	0.014	*	B5_APAPvsB5_VPA	0.03	0.772	
B2vsB4_APAP	0.08	0.263		B1_APAPvsB2_VPA	0.03	0.808	
B2vsB5_APAP	0.58	0.001	**	B1_APAPvsB3_VPA	− 0.04	1.001	
B3vsB4_APAP	0.50	0.001	**	B1_APAPvsB4_VPA	0.05	0.594	
B3vsB5_APAP	0.41	0.001	**	B1_APAPvsB5_VPA	− 0.09	1.001	
B4vsB5_APAP	0.11	0.110		B2_APAPvsB3_VPA	− 0.03	1.001	
B1vsB2_VPA	0.49	0.009	*	B2_APAPvsB4_VPA	0.28	0.019	*
B1vsB3_VPA	0.78	0.001	**	B2_APAPvsB5_VPA	0.24	0.028	*
B1vsB4_VPA	0.41	0.028	*	B3_APAPvsB4_VPA	− 0.10	1.001	
B1vsB5_VPA	0.59	0.002	*	B3_APAPvsB5_VPA	− 0.08	1.001	
B2vsB3_VPA	0.39	0.012	*	B4_APAPvsB5_VPA	− 0.07	1.001	
B2vsB4_VPA	0.88	0.001	**	B2_APAPvsB1_VPA	− 0.04	1.001	
B2vsB5_VPA	0.84	0.001	**	B3_APAPvsB1_VPA	− 0.05	1.001	
B3vsB4_VPA	0.23	0.076		B3_APAPvsB2_VPA	− 0.12	1.001	
B3vsB5_VPA	0.42	0.005	*	B4_APAPvsB1_VPA	− 0.09	1.001	
B4vsB5_VPA	0.84	0.001	**	B4_APAPvsB2_VPA	− 0.11	1.001	
				B4_APAPvsB3_VPA	− 0.07	1.001	
				B5_APAPvsB1_VPA	− 0.00	1.001	
				B5_APAPvsB2_VPA	− 0.06	1.001	
				B5_APAPvsB3_VPA	− 0.01	1.001	
				B5_APAPvsB4_VPA	− 0.02	1.001	

Results showed that 15 out of 20 different possible comparisons appeared as significantly correlated, evidencing the consistency of the metabolic pathways altered upon compound incubated HepG2 cells despite the additional unrelated variation associated to the processing batch. Likewise, the analysis of the correlation between results from functional MetPA from APAP and VPA incubated HepG2 cells showed, as expected, no correlation (*p* value > 0.05) in 22 out of 25 paired comparisons evaluated.

Therefore, despite the frequently unavoidable processing batch effect that influenced metabolite intensities of exposed cells across the different experiments, it was possible to draw consistent and meaningful consequences of metabolic alterations caused by the xenobiotics evaluated by meta-analysis of results from pathway analysis. Thus, upon metabolic pathway analysis, the metabolic pathway alterations deduced remained consistent regardless of the unpreventable causes of variability.

## Discussion

The assessment of hepatotoxic xenobiotic hazards could largely benefit from high-throughput cell metabolomics. This, in addition, would enable to uncover the underlying mechanisms of toxicity thus facilitating new biomarker discovery. The metabolome defines the current metabolic status of a biological system and therefore this *omic* provides the closest information to the phenotypical changes of cells exposed to any insult, providing a real-time picture of the effects induced by a toxic xenobiotic. To perform precise and accurate hazard predictions, high quality and robust metabolic data is required. This, however, is hampered by a series of hardly avoidable sources of variability during the experiments. In a first term, drifting changes in the LC–MS instrumental response known as “within-batch effect” occur and may lead to signal intensity variations decreasing the potential value of data and requiring post-acquisition data correction. This “within-batch effect”, can satisfactorily be corrected, as we previously demonstrated^40^. Besides that, other potential sources of variability arise from the distinct experimental conditions related to the biological system, like for instance, the cell passage number, and to the experimental procedures (i.e., processing and storage time of cellular extracts). Altogether, these factors reduce the relevance of the signals and reduce the reproducibility and repeatability of assays, preventing a proper metabolite profiling and consistent and meaningful biological interpretations. Several studies have already made efforts to address some of these aspects in blood and plasma samples^15–18^. Factors as pre-centrifugation temperature, centrifugation forces, storage temperature, time and tubes, processing time, microclotting and hemolysis were evaluated. Results evidenced that the metabolome is sensitive to a wide range of sample processing factors and reinforced the idea to adhere to established standard operating procedures (SOPs) in metabolomic research. In this work we evaluated these scenarios by treating HepG2 cells with different bioactive substances, as a hepatic model system for hepatotoxicity testing. Cells at different passage number, were incubated with two representative hepatotoxic compounds, namely valproate and acetaminophen, each one known to act by different toxicity mechanisms (valproate interfering with mitochondrial oxidative functions and inducing steatosis, while acetaminophen causes oxidative stress, GSH depletion and irreversible covalent binding to thiol proteins). The concentration of 1000 µM for 24 h was used, because it ensured the occurrence of the toxic phenomena previously mentioned in the absence of a significant cell death. The effects on cell's metabolome were compared with those elicited by citric acid, a non-hepatotoxic compound^27^, at the same concentration to eliminate any possible effect due to the osmolarity rather than to the xenobiotic itself. To cover all possible sources of variations, experiments were designed and conducted on different cell passages, samples were processed on different days and extracts were stored for distinct periods of times, prior to metabolomic analysis to evaluate the influence of all these factors on the metabolome fingerprint obtained and on the hazard estimations derived from such data.

Thus, we performed untargeted metabolite profiling of cell extracts by UPLC-QqTOFMS that provides the detection of polar compounds. The metabolomics MS data generated was initially subjected to QC-SVRC correction to correct within batch effects associated with instrumental drift as previously described^40^. Another real-life situation is the fact that all samples of a large experiment cannot be processed at a time, and so, we compared the metabolomes of samples kept for different storage days prior to the analysis. Thus, within-batch corrected sets of MS data were subjected to non-supervised PCA multivariate data analysis to identify experimental factors that most influence the variation of the metabolic profile. The first two principal components evidenced the strong impact that the processing batch had, clustering samples from different processing batches well separated. ASCA analysis was further performed to quantify the contribution of each of these factors on the global data variability. This data analysis approach allowed us to acknowledge that cellular passage had less relevance than expected, but nevertheless a significant impact on the metabolic profile. The greater source of variation was mostly associated with the processing batch. ASCA also revealed the occurrence of significant impact of compound treatments on cell's metabolome, a fact that was initially underestimated in the PCA, because of the great variability associated with the processing batch.

Given the relevance of the processing batch on the metabolome variance, our efforts were addressed to develop a normalization procedure method to correct this effect. The hierarchical clustering, heatmap and linear regression showed no correlation between metabolite levels and storage days until analysis, contradicting our initial assumption that metabolite changes would be affected in a storage-time dependent manner, and made very difficult data normalization by the processing batch. But interestingly, the analysis of metabolic pathways alterations revealed consistent findings, despite the unavoidable processing batch effect on recorded data. Thus, the way metabolic pathways were altered by the hepatotoxic xenobiotics versus the non-hepatotoxic control, were consistent among the different processing batches. Univariate *t* test analyses between drug and control treatment identified common metabolites altered in the five processed batches of cells treated with each hepatotoxic compound, even though the absolute levels were not significantly altered in each cellular passage/batch combination possible. Yet the direction and the magnitude of the metabolic pathway changes elicited by the xenobiotics were always consistent. Pathway analysis identified six and nine metabolic pathways significantly altered by APAP and VPA respectively in all batches. This reproducibility in the cellular processes is relevant for hazard estimations and was further confirmed by the meta-analysis of pathway analysis results. In addition, the well-separated clustering in the PCA analysis from ASCA factor ‘treatment’ demonstrated that the metabolic profiles obtained upon three different compounds tested, are highly reproducible and consistent independently of the cellular passage and processing batch.

Regarding the metabolic pathways altered upon APAP and VPA treatment, meaningful results were obtained. The liver plays a crucial role in the metabolism of arginine and a correlation of high plasma arginine concentrations with hepatic failure have been previously shown^46^. Interestingly, both xenobiotics discussed here also led to an impact in the arginine biosynthesis.

Specific metabolic pathways appeared altered in each of the hepatotoxic treated conditions. APAP treatment led to an enrichment in the nitrogen metabolism and D-glutamine and D-glutamate metabolism in agreement with a previous study^47^. Increased levels of glutamine may be converted to glutamate enhancing hepatic and mitochondrial GSH that conjugates with APAP metabolized hepatotoxic compound NAPQI for its depletion and safely removal.

VPA hepatotoxicity results from increased oxidative stress caused by a reduction in beta-oxidation^48^. Accordingly, we observed that several lipid-related metabolic pathways appeared consistently altered by VPA treatment such as glycerolipid metabolism, glycerophospholipid metabolism and ether lipid metabolism, in agreement with previous studies^49–51^.

VPA-treated cells had an altered pantothenate and CoA biosynthesis. This pathway is involved in the regulation of VPA-induced hepatotoxic process and has been shown to have hepatoprotective effects upon liver toxicity being a good candidate to treat hepatic disorders and other oxidative stress-related diseases^52^.

Glyoxylate and dicarboxylate metabolism appeared modulated upon VPA treatment and it is also altered in response to several liver diseases^53,54^, suggesting a cellular damage in our tested hepatotoxic conditions.

Several aminoacid related metabolic pathways were significantly altered by either APAP or VPA. An enrichment of histidine metabolism was observed upon APAP treatment. The liver plays a primary role in the metabolism of histamine, the decarboxilation product of histidine, and several studies have described raised levels of histamine in liver diseases^55,56^.

In the case of VPA treatment, alanine, aspartate and glutamate metabolism appeared to be significantly modulated. Interestingly, glutamate metabolism is altered in brain and kidney upon VPA treatment^57–59^. These studies demonstrated that VPA increases glutaminase activity, and our study shows a reduction in glutamine levels upon VPA treatment suggesting that similar molecular mechanisms may be altered by VPA treatment in the liver.

In summary, the experimental workflow used here, was designed to assess the impact of certain experimental factors on the retrieved metabolomics data from HepG2 cells cultured with two hepatotoxic and one non-hepatotoxic compounds. Despite intra batch normalization, metabolome changes due to cell passage (lesser), storage and batch processing had a major influence on raw metabolome intensity signals. However, we have shown that by different approaches with an appropriate data correction with quality control samples as well as by meta-analysis of the altered metabolic pathways, consistent and meaningful conclusions can be made.

## Supplementary Information

Supplementary Information.
