# Supplementary material for: Factors that influence the quality of metabolomics data in in vitro cell toxicity studies: a systematic survey
Source: Sci Rep. 2021 Nov 11;11:22119. doi: 10.1038/s41598-021-01652-1 (PMC8586040; doi:10.1038/s41598-021-01652-1)
Supplement: Supplementary file 1 — Supplementary Information. [file 41598_2021_1652_MOESM1_ESM.pdf]

Supplemental Information

Factors that influence the quality of metabolomics data in *in vitro* cell toxicity studies: A systematic survey

Marta Moreno-Torres, Guillem García-Llorens, Erika Moro, Rebeca Méndez, Guillermo Quintás, José Vicente Castell

Supplemental Experimental Procedures

Metabolomic analysis

Column and autosampler were kept at 40˚C and 4˚C, respectively, and the injection volume was 3 µL. The flow rate was set to 400 µL min^{-1} running a binary mobile phase gradient starting at 99% of mobile phase A (H_2O, 0.1% v/v HCOOH) during 2 min followed by a linear gradient from 1 to 80% of mobile phase B (CH_3CN, 0.1% v/v HCOOH) during 8 min, from 80% to 98% v/v of mobile phase B in 0.1 min, 98% of mobile phase B was maintained for 0.9 min; return to initial conditions was achieved in 0.1 min and were maintained for a total run time of 15 min.

Full scan MS-data from 50 to 1200 m/z was collected on an Agilent 6550 Spectrometer iFunnel quadrupole time-of-flight (QTOF) MS system (Agilent Technologies). Samples were analyzed using positive and negative electrospray ionization (ESI) in separate batches. Between each mode, the instrument was cleaned and calibrated according to the manufacturer's instructions. MS data was acquired at a scan frequency of 5 Hz using the following parameters: gas T, 200 °C; drying gas, 14 L/min; nebulizer, 37 psi; sheath gas T, 350 °C; sheath gas flow, 11 L min^{-1}. Mass reference standards were introduced into the source for automatic MS spectra recalibration during analysis via a reference sprayer valve using the 149.02332 (phthalic anhydride), 121.050873 (purine), and 922.009798 (HP-0921) *m/z* in ESI+, and 119.036 *(purine*) and 966.0007 ([HP-0921+HCOOH-H]-) m/z in ESI-, as references.

The analytical batch included 239 samples in randomized order, 29 quality control (QC) samples (1 every 10 samples). 3 Blanks and 3 QCs were run at

the beginning of the sequence for the generation of the inclusion list, followed by 9 QCs for MS/MS acquisition and 3 control blanks, 2 media blanks, and 2 QCs before the first sample. The last QC sample from the sequence was followed by 3 media blanks and 2 control blanks. QCs samples were used to monitor LC-MS system performance, correct within-batch effects, and identify unreliable features [1]. QCs were initially injected for system conditioning and auto MS/MS data dependent acquisition method with the following inclusion *m/z* precursor ranges: 50–200, 200–300, 300–400, 400–500, 500–600, 600–700, 700–800, 800-900, and 900–1200 from 50 to 1200 using, in all replicates, centroid mode at a rate of 5 spectra/s in the extended dynamic range mode (2 GHz). After that, and in order to increase the coverage of fragmented features, targeted dynamic iterated DDA was performed in which MS/MS spectra were acquired using an inclusion list of pre-annotated features after the injection of 3 blanks and 3 QCs during system conditioning, as described elsewhere [2]. In this case, LC-MS features were added to the ESI+ inclusion list if they were not detected in blanks and could be (pre) annotated as a $[M+H]^+$, $[M+Na]^+$, $[M+NH_4]^+$, $[M+H+Na]^{+2}$, $[M+K]^+$, $[M+H+K]^{+2}$, $[M+H+CH_3CN]^+$, $[M+H+2CH_3CN]^+$, $[M+Na+CH_3CN]^+$, $[M+2Na-H]^+$, $[2M+H]^+$, $[2M+Na]^+$, $[2M+K]^+$, $[2M+NH_4]^+$, $[2M+H+CH_3CN]^+$, $[2M+Na+CH_3CN]^+$, or $[M+H-H_2O]^+$ adducts of, at least, one of the 95688 metabolites included in the HMDB with a m/z accuracy error <20 ppm. In ESI-, the list of potential adducts included $[M-H]^-$, $[M+Cl]^-$, $[M+H_2O-H]^-$, $[M+2Na-H]^-$, $[M+K-H]^-$, $[2M-H]^-$, $[2M+HCOOH-H]^-$, and $[M+HCOOH-H]^-$. Collision energy was set to 20 V, MS/MS fragmentation with automated selection of five precursor ions per cycle and an exclusion window of 0.15 min after two consecutive selections of the same precursor.

Metabolite annotation

Briefly, the annotation algorithm determines whether each feature with available MS/MS spectra can be pre-annotated with an m/z accuracy error below 20 ppm, as a $[M+H]^+$, $[M+Na]^+$, $[M+H+Na]^+$, $[M+K]^+$, $[M+H+K]^{+2}$, $[M+H+CH_3CN]$, $[M+H+2(CH_3CN)]^+$, $[M+Na+CH_3CN]^+$, $[M+2Na-H]^+$, $[2M+H]^+$, $[2M+Na]^+$, $[2M+K]^+$, $[M+NH_4]^+$, $[2M+H+CH_3CN]^+$, or $[2M+Na+CH_3CN]^+$ adducts in ESI+, or as a $[M-H]^-$, $[M+Cl]^-$, $[M+H_2O-H]^-$, $[M+2Na-H]^-$, $[M+K-H]^-$,

$[2M-H]^-$, $[2M+FA-H]^-$, $[2M+CH_3COOH-H]^-$, or $[M+FA-H]^-$ adduct in ESI-, of at least, one metabolite included in the MS/MS database. If so, the closest experimental MS/MS spectrum is matched against the spectral database. For each potential match, a spectral dot product (*dp*) and a reverse dot product (*rdp*) are calculated as described elsewhere [3], using in this study m=1.2 and n=0.9 for *dp* and *rdp*, respectively. The calculation of the *rdp* only included ions present in both the experimental and reference spectrum. Then, the geometric mean of the *dp* and *rdp* is calculated and the identity of the metabolite with the largest *mean dot product* is stored. Further parameters included: a minimum number of matching ions in the experimental and reference spectra equal to 3; absolute and relative intensity thresholds in the MSMS spectra of 0.01% of the base peak and 200 AU; minimum *mean dot product:* 0.25. Metabolite annotation using LipidBlast was carried out using LipiDex [4] using 0.01 Da tolerances in both MS (precursor) and MS^2 (fragment) data and the 'LipidBlast Acetate' library. When an LC-MS feature was annotated, features included in the same pseudospectrum (i.e., same CAMERA *pcgroup*), also detected in the experimental and reference MS^2 spectrum (with *m/z* accuracy error<20 ppm, and an intensity above an absolute and/or relative threshold), were labeled as fragments of the annotated metabolite.

Supplementary Figures

Figure S1

a

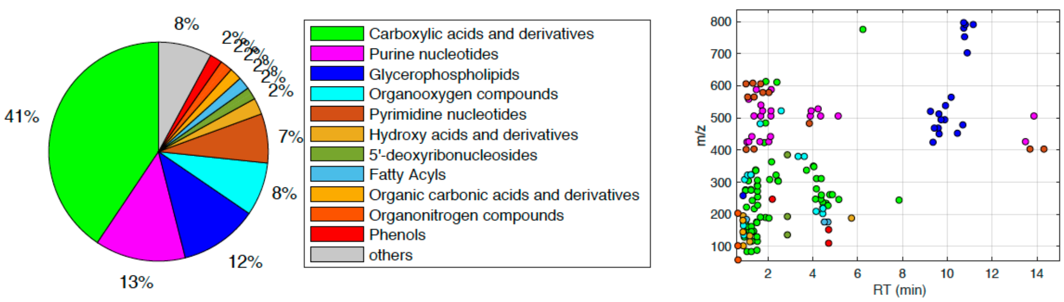

b

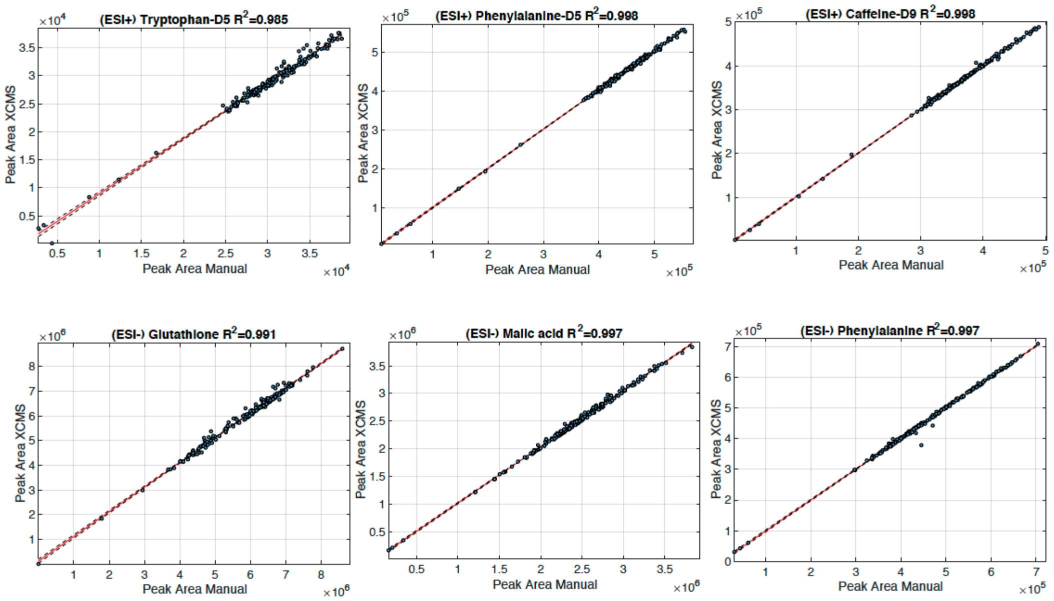

Figure S1. Peak table generation quality control and distribution of LC-MS annotated features and classes retained after data pre-processing. (a) Pie chart shows the percentage of features annotated to each class in both ionization modes after data pre-processing and clean up. Only classes with more than 2 annotated features were included. Right panel shows the distribution of the identified signals according to their m/z and retention time (RT). (b) Dispersion diagram that represents the area values from the XCMS versus the area values calculated with the Agilent MassHunter Profinder for the indicated internal standards (Phenylalanine-D_5, Caffeine-D_9 and Tryptophan-D_5) or endogenous metabolites (Glutathione, Malic acid and

Phenylalanine). The linear regression lines and the R-squared values are shown in all graphs.

Figure S2

a **b**

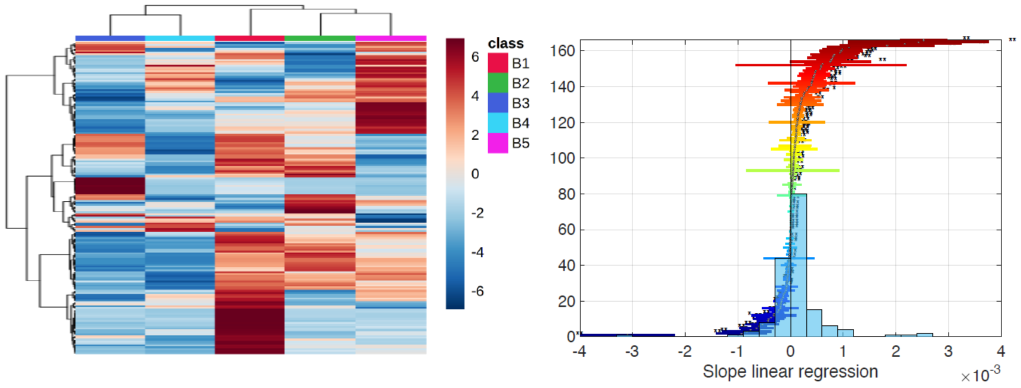

Figure S2. No linear correlation between metabolite intensities and storage time. a) Heatmap analysis of the 166 metabolites of the clustering according to the ASCA factor 'batch' including Batch 1 to 5. Each column represents the group average for all samples from each batch and each row represents a different metabolite. Color code inside the heatmaps indicates the intensity of the annotated metabolite; blue and red express the lowest and the highest abundances, respectively. Clustering: Ward’s linkage algorithm with Euclidean distances. Heatmap analysis was performed with MetaboAnalyst and no data transformation or data scaling was performed.

b) Values of the slope (dot) and standard deviations (color bars) from the linear regressions calculated between metabolite intensities and storage time. Significant values (p-value<0.05) are shown with an asterisk. Histogram shows the number of metabolites with different slopes for the linear regression.

Supplementary Tables

Acetaminophen	Valproate
D-Glutamine and D-glutamate metabolism	Alanine, aspartate and glutamate metabolism
Nitrogen metabolism	Ether lipid metabolism
Arginine biosynthesis	Glycerophospholipid metabolism
Aminoacyl-tRNA biosynthesis	Glyoxylate and dicarboxylate metabolism
Arginine and proline metabolism	Glycine, serine and threonine metabolism
Histidine metabolism	Pentose phosphate pathway
	Arginine biosynthesis
	Glycerolipid metabolism
	Pantothenate and CoA biosynthesis

Table S1. Significant pathways altered upon acetaminophen or valproic acid versus citric acid treated HepG2 cells. Pathway Analysis from MetaboAnalyst was performed to identify significant pathways (p-value<0.05) commonly altered in all five batches. Data was log transformed and autoscaled.

References

1. Sanchez-Illana, A., et al., *Model selection for within-batch effect correction in UPLC-MS metabolomics using quality control - Support vector regression.* Anal Chim Acta, 2018. **1026**: p. 62-68.
2. Kuligowski, J., et al., *Intra-batch effect correction in liquid chromatography-mass spectrometry using quality control samples and support vector regression (QC-SVRC).* Analyst, 2015. **140**(22): p. 7810-7.
3. Stein, S.E. and D.R. Scott, *Optimization and testing of mass spectral library search algorithms for compound identification.* J Am Soc Mass Spectrom, 1994. **5**(9): p. 859-66.

4. Hutchins, P.D., J.D. Russell, and J.J. Coon, *LipiDex: An Integrated Software Package for High-Confidence Lipid Identification.* Cell Syst, 2018. **6**(5): p. 621-625 e5.
